# Managerial strategies and policies – Analysis of patient satisfaction based on demographic data

**DOI:** 10.25122/jml-2018-0065

**Published:** 2018

**Authors:** Gabriel Radu, Maria Radu, Andrei Condurache, Victor Lorin Purcărea

**Affiliations:** 1. Carol Davila University of Medicien and Pharmacy; 2. Universitatea de Medicina si Farmacie Carol Davila; 3. Universitatea de Medicina si Farmacie Carol Davila Facultatea de Medicina

**Keywords:** satisfaction, demographic, managerial strategies

## Abstract

Service quality of healthcare organizations is more and more determined by the patients’ satisfaction data. Therefore, the fairness of this comparison raises questions. The factors that could affect it are represented by the demographic characteristics of patients but also the institutional characteristics of the healthcare organization. In this study, we will analyze the demographic factors. 150 questionnaires were analyzed, completed by the patients of an obstetrics-gynecology clinic in order to complete an observer study. Observing the differences between the demographic aspects of the patients, we decided to analyze them using a few satisfaction scores that were built on the answers received on the basis of the questionnaire. Each satisfaction score was attributed to a category of satisfaction surveyed between patients, and its interpretation was based on the answers received and the number of questions. The analysis of the results has shown the existence of many correlations observed in the study, based on the built-up scores, the demographic data of the patients (background, age category, level of education, monthly income level, civil status, gynecological consultation objective, sex of the gynecologist) and the way the patients were informed and their general impression.

## Introduction

Service quality of healthcare organizations is more and more determined by the patients’ satisfaction data. Therefore, the fairness of this comparison raises questions [1]. The factors that could affect it are represented by the demographic characteristics of patients but also the institutional characteristics of the health care organization. In this study we will analyze the demographic factors [2,3]. Satisfaction should be a predictor for evaluating health care services. Different studies show that older users are more frequently satisfied with life while people with below average self-perceived health reported lower life satisfaction [4]. Louise, Matthews and Stones consider that consumers expect understanding, respect, thorough information, access, correctitude, competence and good results [5]. The goal of this study is to examine if the patient’s satisfaction scores are correlated to his demographic factors.

## Methods and materials

Starting from the data obtained after analyzing 150 questionnaires completed by patients of an obstetrics-gynecology clinic in Bucharest, an observational study was carried out. The questionnaire includes questions about the demographic data of the patients (the environment of origin, age, level of education, level of income, civil status, the reason for the gynecological consultation and the sex of the gynecologist), questions about the way the patients were informed and their general impression of the gynecology clinic, but also questions grouped on 5 categories: the orientation towards the healthcare consumer, a good employer, a cost-effective and financially viable organization, the quality of services and social responsibility. The questions have 5 possible answers: a. Total disagreement, b. Disagreement, c. Neither disagreement nor agreement, d. I agree, e. I fully agree.

The data obtained was collected, analyzed and presented using IBM SPSS Statistics 19 and Microsoft Office Excel 2013, and Microsoft Office Word 2013. The quantitative variables were tested for distribution using the Shapiro-Wilk test, Mann-Whitney U or Kruskal Wallis H, and the existing correlations were demonstrated using the Spearman’s Rho correlation coefficient. The qualitative variables were tested using the Pearson Chi-Square test.

## Results

All 150 (100%) of patients completed the study. Observing their demographic data, it was determined that most of them came from the urban area (59.3%), they had higher education (49.3% – university studies, 14% – postgraduate studies), they had a high monthly income (52% – over RON 3,000) and are currently married (54%) (Table 1).

Observing these differences, between the demographic aspects of the patients, we decided to analyze them using some satisfaction scores that were built using the answers received on the questionnaire and that are presented in Table 2.

Each satisfaction score was attributed to a category of satisfaction surveyed between patients, and its interpretation was based on the answers received and the number of questions.

**Table 1:** Distribution of patients depending on the demographic data

Demographic data		
*Environment of origin*	n	%
Urban	89	59.3%
Rural	61	40.7%
***Level of education***		
Secondary school studies	21	14%
High school studies	34	22.7%
University studies	74	49.3%
Postgraduate studies	21	14%
***Monthly income***		
<1200 RON	11	7.3%
1300-1500 RON	7	4.7%
1600-2000 RON	8	5.3%
2100-2500 RON	12	8%
2600-3000 RON	34	22.7%
>3000 RON	78	52%
***Civil status***		
Unmarried	56	37.3%
Married	81	54%
Widow	13	8.7%

**Table 2:** Satisfaction scores used in the study

Name	Description
**SCOR_CONS**	Evaluation score regarding the orientation towards the consumer of medical services
**SCOR_ANG**	Evaluation score regarding the appreciation of the gynecology clinic as a good employer
**SCOR_ORG**	Evaluation score on the assessment of the gynecological organization as a cost-effective and financially viable organization
**SCOR_QUAL**	Evaluation score regarding service quality
**SCOR_RESP**	Evaluation score regarding social responsibility

We considered that there were five possible answers for each question, the options being: I completely disagree – 0 points, I disagree – 1 point, I am undecided – 2 points, I agree – 3 points and I completely agree – 4 points. Classifying a group of questions in a category and adding up the scores we will get a satisfaction score that follows a certain aspect.

Satisfaction level categories for each score will be described below in Table 3.

**Figure 1:**
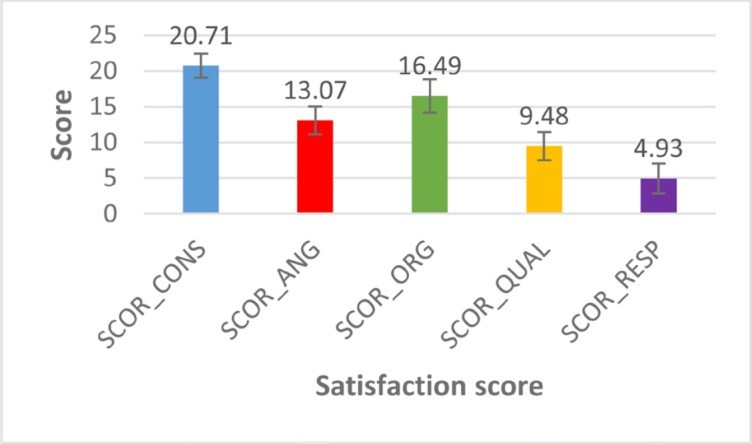
The mean value of satisfaction scores analyzed in the study participants.

Each satisfaction score was attributed to the satisfaction category determined between the patients. On the studied lot, these scores were calculated and the results are presented in Figure 1.

**Table 3:** Satisfaction categories correlated with the scores

Satisfaction category	Inclusion
**Totally dissatisfied**	**0-4** points for SCOR_CONS; SCOR_ANG;
	**0-5** points for SCOR_ORG;
	**0-2** points for SCOR_QUAL; SCOR_RESP
**Partially dissatisfied**	**5-9** points for SCOR_CONS;
	**5-8** points for SCOR_ANG;
	**6-10** points for SCOR_ORG;
	**3-6** points for SCOR_QUAL
	**3-4** points for SCOR_RESP
**Neutal**	**10-14** points for SCOR_CONS;
	**9-12** points for SCOR_ANG;
	**11-16** points for SCOR_ORG;
	**7-10** points for SCOR_QUAL
	**5-7** points for SCOR_RESP
**Partially satisfied**	**15-19** points for SCOR_CONS;
	**13-16** points for SCOR_ANG;
	**17-22** points for SCOR_ORG;
	**11-13** points for SCOR_QUAL
	**8-10** points for SCOR_RESP
**Very satisfied**	**20-24** points for SCOR_CONS;
	**17-20** points for SCOR_ANG;
	**23-28** points for SCOR_ORG;
	**14-16** points for SCOR_QUAL
	**11-12** points for SCOR_RESP

As it can be seen in Figure 1, the results show that patient satisfaction regarding the orientation towards the healthcare consumer is 20.71 ± 1.692 points, which is a **very satisfying** level for this level of satisfaction.

Patient satisfaction regarding the appreciation of the gynecological organization as a good employer is also rated at 13.07 ± 1.948 points, which is a **partial satisfaction** for this level of satisfaction.

Patient satisfaction regarding appreciation of the gynecological organization as a cost-effective and financially viable organization is evaluated at 16.49 ± 2.337 points, which is a **neutral** level for this level of satisfaction.

Patient satisfaction with the quality assessment of the gynecologist services is evaluated at 9.48 ± 1.979 points, which is a **neutral** level for this level of satisfaction.

Patient satisfaction with the social responsibility of the organization is evaluated at 4.93 ± 2.088 points, which is a **partial dissatisfaction** level for this level of satisfaction.

**Figure 2:**
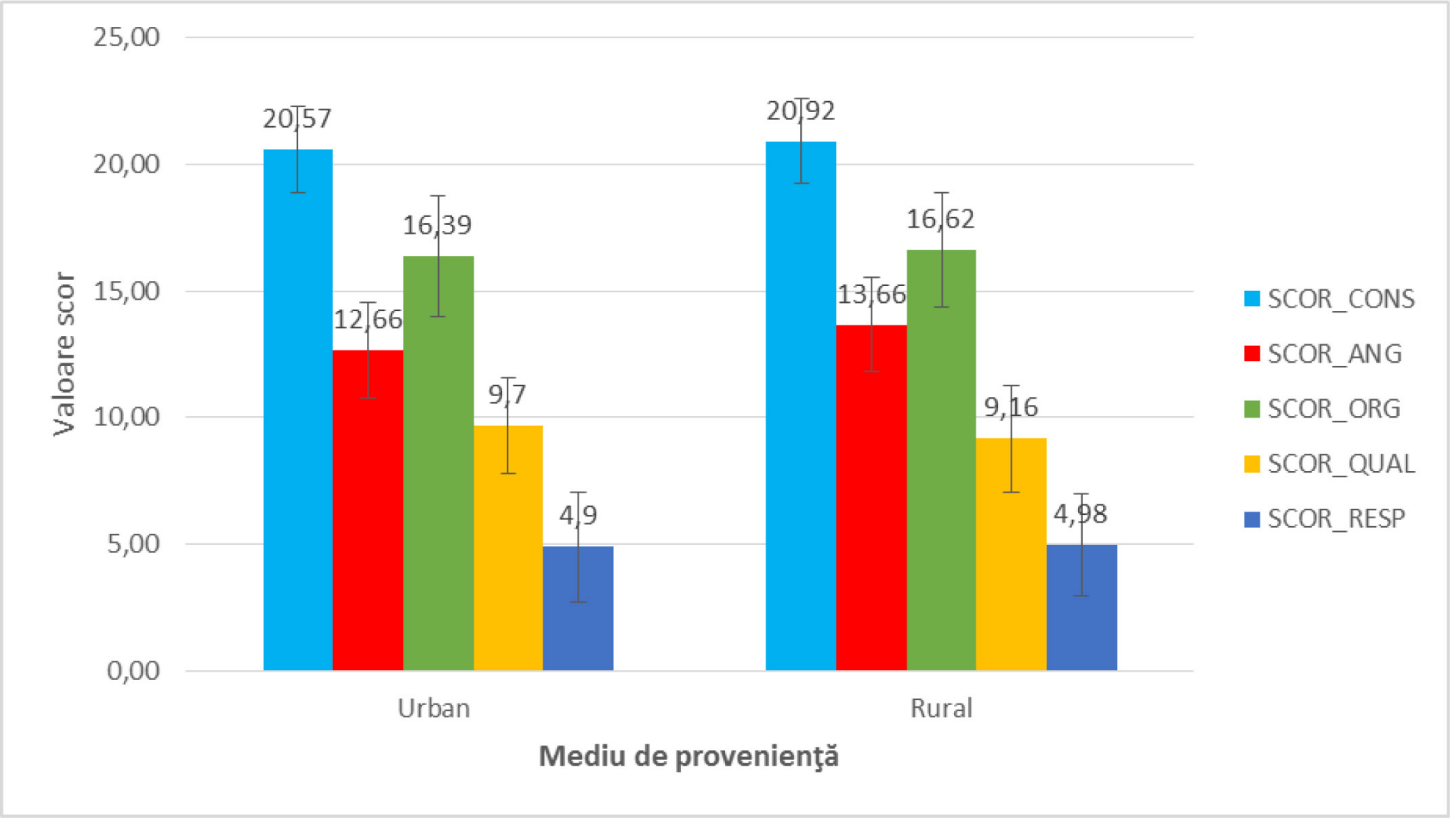
The mean value of satisfaction scores analyzed in the study participants reported to the background.

**Figure 3:**
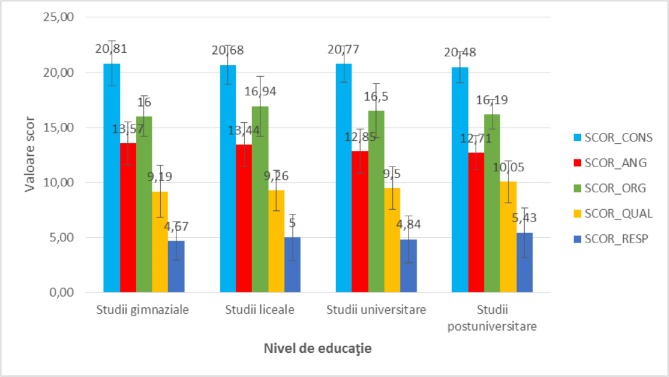
The mean value of satisfaction scores analyzed in study participants reported to the level of education.

**Figure 4:**
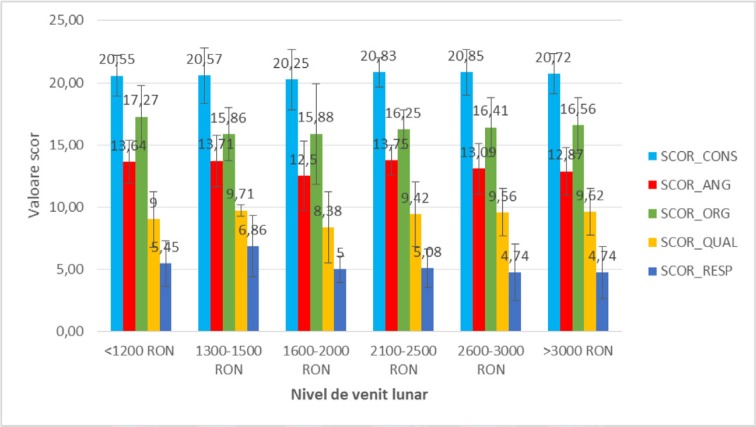
The mean value of satisfaction scores analyzed in the study participants reported to the monthly income.

**Figure 5:**
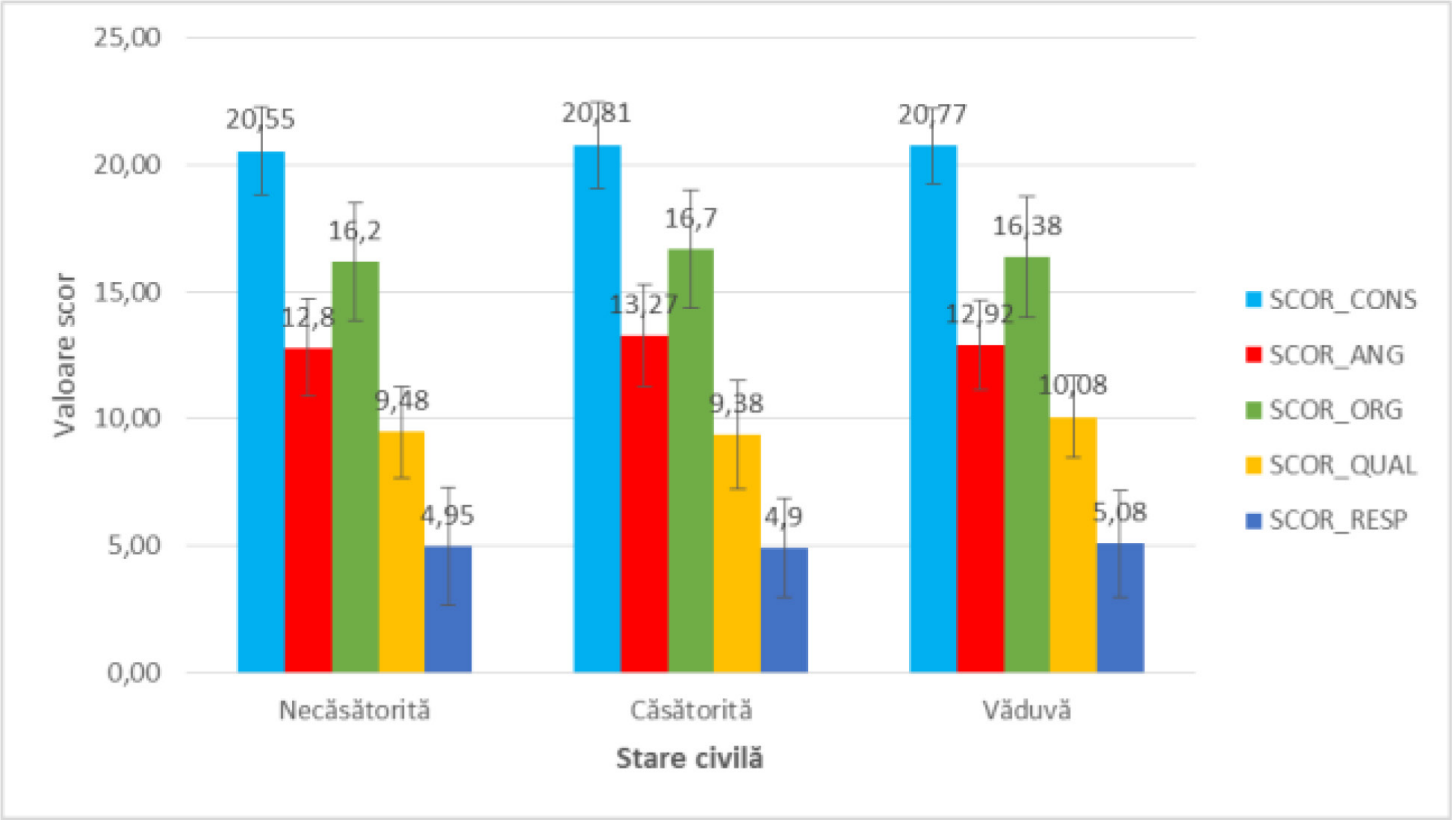
The mean value of satisfaction scores analyzed in study participants reported to the civil status.

It was decided to analyze these scores against demographic factors, as outlined in Figures 2,3,4,5.

Regarding the **environment of origin**, satisfaction scores among urban and rural patient groups did not differ significantly statistically, as shown in Figure 2 (p = 0.137 – SCOR_CONS, p = 0.975 – SCOR_ORG, p = 0.109 – SCOR_QUAL, p = 0.958 – SCOR_RESP), with the exception of the score that evaluated the gynecological organization as a good employer (p = 0.002 – SCOR_ ANG). The demonstrated correlation shows that rural patients have higher satisfaction in this regard than urban patients (p = 0.002, R = 0.252).

Regarding the **level of education**, the satisfaction scores among the groups of patients with secondary school, high school, university and postgraduate studies did not differ significantly statistically, according to Fig. 3 (p = 0.746 – SCOR_CONS, p = 0.259 – SCOR_ANG, p = 0.315 – SCOR_ORG, p = 0.460 – SCOR_QUAL, p = 0.626 – SCOR_RESP), so that the level of education did not alter the perception of patients regarding the analyzed aspects.

Regarding the **monthly income level**, the satisfaction scores among the groups of patients with secondary school, high school, university and postgraduate studies did not differ significantly statistically, according to Fig. 4 (p = 0.989 – SCOR_CONS, p = 0.343 – SCOR_ANG, p = 0.819 – SCOR_ORG, p = 0.557 – SCOR_QUAL, p = 0.389 – SCOR_RESP), so that the monthly income level did not alter the perception of patients on the issues under consideration.

Regarding the **civil status**, the satisfaction scores among the groups of patients with secondary school, high school, university and postgraduate studies did not differ significantly statistically, according to Fig. 5 (p = 0.764 – SCOR_CONS, p = 0.275 – SCOR_ANG, p = 0.660 – SCOR_ORG, p = 0.329 – SCOR_QUAL, p = 0.990 – SCOR_RESP), so that the civil status did not alter the perception of patients on the issues under consideration.

**Figure 6:**
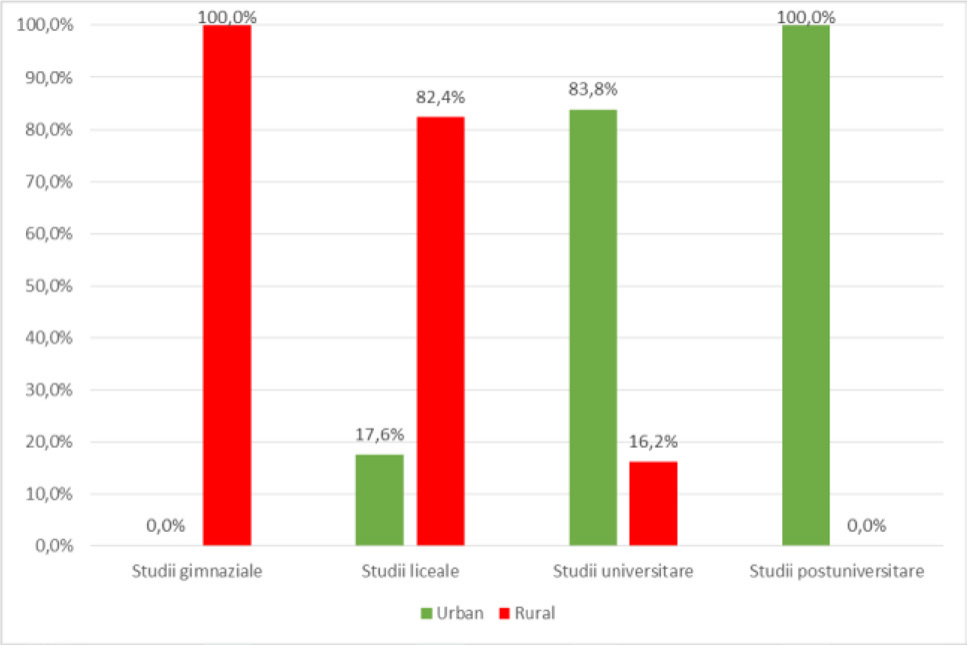
Distribuţia pacientelor în funcţie de mediul de provenienţӑ şi nivelul de educaţie

**Figure 7:**
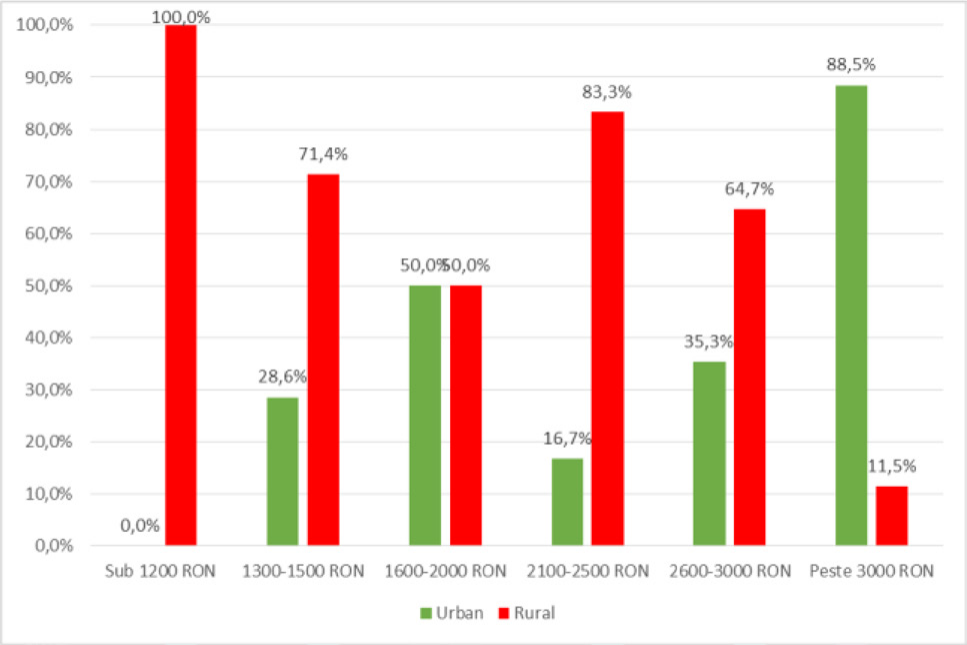
Distribution of patients according to the background and monthly income level.

**Figure 8:**
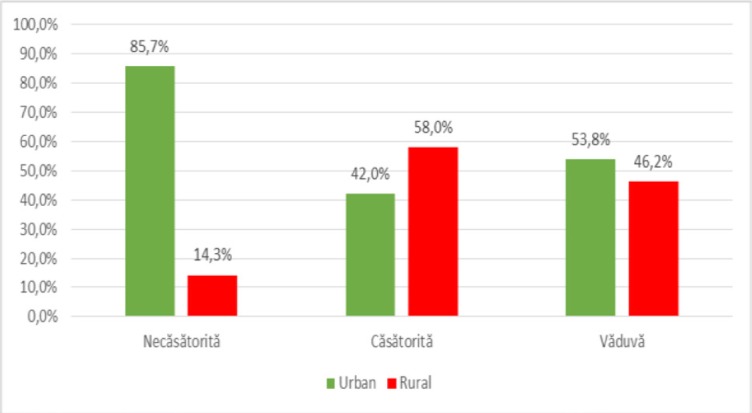
Distribution of patients according to the background of origin and civil status.

**Figure 9:**
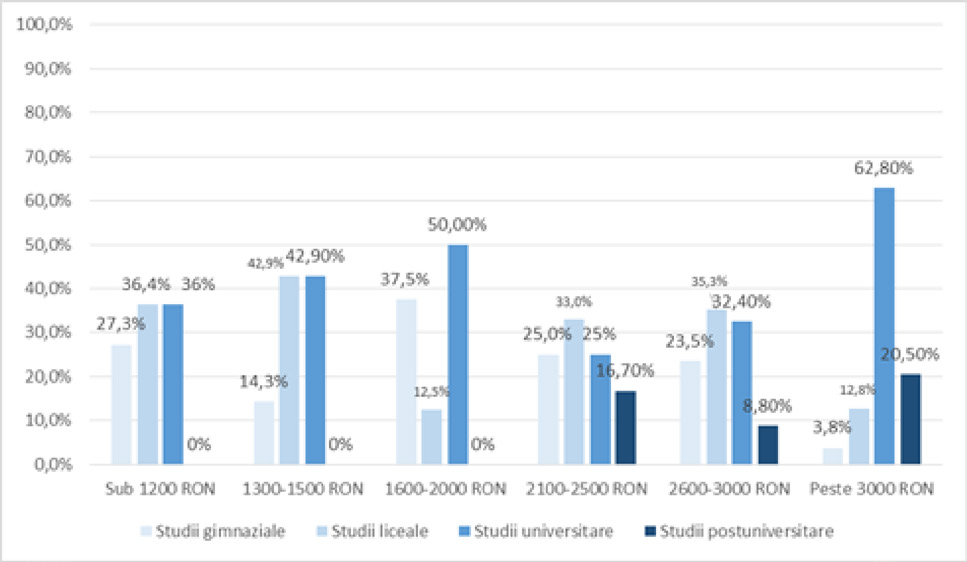
Distribution of patients according to the education level and income level.

**Figure 10:**
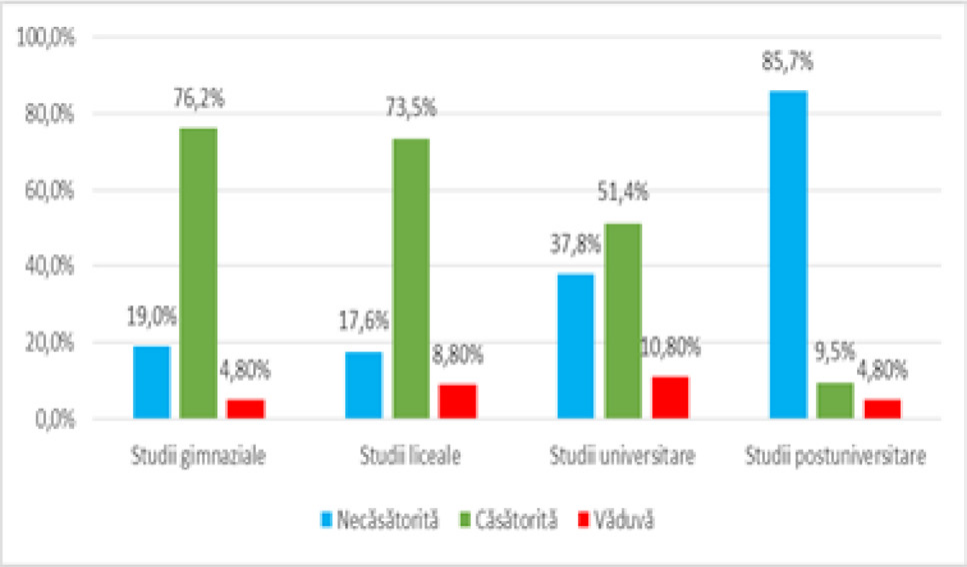
Distribution of patients according to the education level and civil status.

Subsequent analysis between demographic data demonstrated the existence of statistically significant differences, as shown in Figures 6, 7, 8, 9, 10.

The distribution of patients by the **environment of origin** and **level of education**, as outlined in Figure 6, showed that there is a significant difference (p <0.001) between urban patient groups and rural patients and their levels of education (secondary school, high school studies, university studies, postgraduate studies) in the sense that secondary school students are predominant in rural areas (100% – secondary, 82.4% – high school) and patients with university or postgraduate studies are predominantly urban (83.8% – university studies, 100% – postgraduate studies) according to the significant and negative correlation (p < 0.001, R = -0.726).

The distribution of patients by the **background** and the **monthly income level**, as shown in Figure 7, showed a significant difference (p <0.001) between urban patient groups and rural patients and their monthly income levels (below 1,200 RON, 1300–1500 RON, 1600–2000 RON, 2100–2500 RON, 2600–3000 RON or over 3000 RON) meaning that patients with income below RON 1200 or RON 1300–1500 are predominant in rural areas (100% – income under RON 1200, 71.4% – income between 1300–1500 RON) and the patients with over RON 3,000 are predominant from the urban area (88.5%) according to the significant and negative correlation (p <0.001, R = -0.543), indicating an inclination of high-income patients to the urban environment and those with low income to rural areas.

The distribution of patients by **environment of origin** and **civil status**, as shown in Figure 8, showed that there was a significant difference (p <0.001) between urban patient groups and rural patients and their civil status (unmarried, married or widows) in the sense that unmarried patients are predominant in urban areas (85.7%) and married patients are predominantly rural (58%) according to the significant and positive correlation (p <0.001, R = 0.342).

The distribution of patients according to the **level of education** and **income level**, as shown in Figure 9, showed that there was a significant difference (p = 0.002) among groups of patients with secondary, high school, university or postgraduate studies and their level of income below RON 1,200, 1300–1500 RON, 1600–2000 RON, 2100–2500 RON, 2600–3000 RON or over 3000 RON) in the sense that the patients with income below RON 1200 predominantly have secondary (27.3%) or high school studies (36.4%), as well as between 1300–1500 RON (secondary school education – 17.3%, high school education – 42.9%), while the patients with over RON 3,000 have university studies (62.8%) or postgraduate studies (20.5%), according to the significant and positive correlation (p <0.001, R = 0.346).

The distribution of patients according to **education** and **marital status**, as shown in Figure 10, showed that there was a significant difference (p <0.001) among groups of patients with secondary school, high school, university and postgraduate studies and marital status (unmarried, married or widows) in the sense that the patients with secondary school studies are predominantly married (76.2%), a similar situation in the case of high school (73.5% – married) while the postgraduate students are predominantly unmarried (85.7%), according to the significant and negative correlation (p <0.001, R = -0.290).

The analysis of the results has revealed the existence of many correlations observed in the study, starting from the built-up scores, the demographic data of the patients and the way the patients were informed and their general impression of the gynecology clinic: • Assessment Score on orientation towards the healthcare consumer (SCOR_CONS); • Evaluation score to evaluate the gynecological organization as a good employer (SCOR_ ANG); • Evaluation score on the appreciation of the gynecological organization as a cost-effective and financially responsible organization (SCOR_ORG); • Service Quality Score (SCOR_QUAL); • Social Responsibility Assessment Score (SCOR_RESP)

## Discussions

It has been noticed that there are no significant differences between groups based on the environment of origin in terms of satisfaction with the gynecological organization’s orientation towards the healthcare consumer, the appreciation of the gynecological organization as a cost-effective and financially viable organization, satisfaction with the service quality and the satisfaction of assessing the social responsibility of the gynecological organization, perhaps due to a unanimous opinion both for urban and rural patients.

Regarding the satisfaction with the appreciation of the gynecological organization as a good employer, urban patients have a lower level of satisfaction than rural patients.

Observing the level of education of the patients studied, the satisfaction regarding the orientation of the gynecological organization towards the healthcare consumer, the satisfaction regarding the appreciation of the gynecological organization as a good employer, the satisfaction of appreciating the gynecological organization as a cost-effective and financially viable organization, satisfaction with the quality of the gynecologist’s services and satisfaction with the assessment of the social responsibility of the gynecological organization, there are no significant differences between groups, probably due to the fact that, compared to the level of education, the quality of the medical services offered was the same and therefore did not change the perception of the patients about these issues.

In case of analyzing the patients’ response according to the monthly income level and the civil status to the satisfaction regarding the orientation of the gynecological organization towards the consumer, the satisfaction regarding the appreciation of the gynecological organization as a good employer, the satisfaction regarding the appreciation of the gynecological organization as a cost-effective from the financial point of view, the satisfaction of the quality of the gynecologist’s services and the satisfaction regarding the social responsibility assessment of the gynecological organization, there were no significant differences between the groups, to the same extent that there were no differences regarding the level of education related to the monthly income, probably also due to the quality of the medical services provided by the gynecologist and the gynecological organization, whose perceptions have been modified by the civil status of the patients.

It has also been noticed that urban patients often have university and postgraduate studies compared to rural patients who are more likely to have secondary or high school education. It can also be observed that urban patients have a high monthly income comparable to that of rural patients. Regarding the environment of origin and the marital status of patients, it can be seen that urban patients are more often unmarried compared to those in rural areas that are more often married.

It can also be noticed that lower-income patients have more frequent secondary school studies.

It has also been noticed that patients with secondary school or high school studies are more frequently married while patients with university or postgraduate studies are more often unmarried.

This study sought to track and achieve results among patients benefiting from gynecological services, their demographics and satisfaction levels. It has been observed in this study that the home environment has significant importance in differentiating patients, both in terms of adduction level, monthly income level, and satisfaction levels.

In order to benefit from increased effectiveness in the future, this study would require an extensive batch of patients, so that the study could better extrapolate the data and differences from the study to the global population and also corroborate these data with current literature information.

## Conflict of Interest

The authors confirm that there are no conflicts of interest.
